# Evaluation of the risk factors of dental caries in children with very low birth weight and normal birth weight

**DOI:** 10.1186/s12903-020-01372-4

**Published:** 2021-01-07

**Authors:** Romana Koberova, Vladimira Radochova, Jana Zemankova, Lenka Ryskova, Zdeněk Broukal, Vlasta Merglova

**Affiliations:** 1 Department of Dentistry, Faculty of Medicine Charles University, University Hospital in Hradec Kralove, Sokolska 581, 50005 Hradec Králové, Czech Republic; 2 Department of Paediatrics, Faculty of Medicine Charles University, University Hospital in Hradec Kralove, Hradec Králové, Czech Republic; 3 Department of Microbiology and Clinical Immunology, Faculty of Medicine Charles University, University Hospital in Hradec Kralove, Hradec Králové, Czech Republic; 4 Institute of Clinical and Experimental Dental Medicine, 1st Faculty of Medicine, Charles University and General University Hospital in Prague, Prague, Czech Republic; 5 Depatrment of Dentistry, Faculty of Medicine Charles University, University Hospital in Pilsen, Pilsen, Czech Republic

**Keywords:** Infants, Very low birth weight, Pre-term birth, Dental caries, Risk factors

## Abstract

**Background:**

Health problems of premature infants can affect both general and oral health. The enamel defects, poor dietary and oral hygiene habits may predispose these children to dental caries. This study was conducted to assess the impact of very low birth weight and prematurity on caries risk in early childhood.

**Methods:**

The study cohort consists of 189 of one year old infants. Anamnestic data were obtained from hospital records, feeding practice, bed-time sugar drinks and oral hygiene onset from questionnaires. Saliva samples of children and their mothers were collected for the detection of cariogenic pathogens.

**Results:**

VLBW newborns had significantly shorter gestation age (29.6 vs. 38.8)) and lower mean birthweight (1124 g vs 3315 g) compared to NBW ones (p < 0.0001). Statistical significance has been found in the presence of early morbidity (p < 0.0001) and regular medication intake (p = 0.007). VLBW children got more frequently sweetened drinks during the day and night (p = 0.007). Regular oral hygiene practice was more frequent in full term group (p = 0.002). There was statistical difference in the presence of enamel hypoplasia in VLBW children (p = 0.033) but no statistical difference in the presence of hypomineralization (p = 0.0736) in comparison to NBW individuals. Proportional representation and count of *S. mutans* did not reveal statistical difference neither in both groups of children (p = 0.484) nor in both groups of mothers (p = 0.385).

**Conclusions:**

The study confirmed anamnestic and medical differences between both groups. The proportional representation and count of *S. mutans* did not reveal statistical difference neither in VLBW and NBW children, nor in their mothers.

## Introduction

Low birth weight (LBW) is defined as a birth weight of newborn of 2500 g or less. Very low birth weight (VLBW) is defined as a birth WEIGHT of newborn of 1500 g or less [1]. VLBW is frequently associated with pre-term birth when gestation age less than 37 weeks. Premature and both LBW and VLBW infants have a shorter prenatal period and are at risk to various perinatal and neonatal complications and developmental problems that can affect their general growth and progress during infancy and throughout childhood [2]. LBW is a public health issue because it is closely related to infant mortality and to an increased risk of early and late morbidity, like cerebral palsy, seizure disorders, severe mental retardation, and respiratory tract infections. Premature birth may also affect the developmental and cognitive profiles, IQ scores and perceptual abilities having impact on the child´s behaviour and cooperation during oral health care, dental examination and treatment [2].

Pre-term delivery and LBW can cause severe dental problems like hypomineralization and hypoplasia in both primary and permanent dentition [3, 4]. Last trimester of pregnancy is critical for the optimal incorporation of minerals into enamel matrix of primary teeth. Therefore enamel of pre-term children is more porous and less resistant to acidic oral environment. Enamel hypoplasia is more frequently seen on primary incisors like semi-lunar cut of incisal edge [3]. Rough surface of hypo plastic enamel promote plaque formation, and *Streptococcus mutans* adhesion what may lead to quick progression of caries attack on primary teeth.

The enamel defects with other risk factors as feeding problems, frequent intake of sugar containing medication, oral hygiene habits, early transmission of cariogenic bacteria and behavioural disorders predispose these children to dental caries. Davenport et al. [5] have reported a greater sugar intake, and consequently greater number of dental caries in premature and VLBW children when to compare with full term born children. The recent dental literature has focused on the microbial colonization of healthy full-term infants and their mothers or caregivers. The literature shows, that the microbial colonization and transmission of cariogenic microbes to the oral cavity of infants and the role of *Streptococcus mutans* are considered as the risk factor of early childhood caries [6, 7]. However, the oral microbial acquisition in pre-term, VLBW infants has not been adequately investigated.

The aim of this study was to identify risk factors for dental caries of one year old pre-term, VLBW children and to assess the impact of very low birth weight and prematurity on caries risk in early childhood. We hypothesized that premature, VLBW infants will have different level of cariogenic bacteria in their oral cavity due to differences in the immune system.

## Methods

This prospective controlled study is a part of long-term research project supported by the Ministry of Health of the Czech Republic and performed at the Department of Dentistry and Neonatology Faculty of Medicine and University Hospital in Pilsen and Hradec Kralove. Ethical approval for the investigation was obtained from the Research Ethic Committees of both institutions. The study was conducted in accordance with the Helsinki Declaration of children´s rights 1975 (revised 1983). The study group infants were selected from the Departments of Neonatology, University Hospital in Pilsen and Hradec Kralove. The control group infants were selected similarly from the Department of Paediatric Dentistry of both institutions. The laboratory tests were performed at the Department of Microbiology and Immunology, University Hospital in Hradec Kralove and the Research Institute of Clinical and Experimental Dentistry, University Hospital in Prague. The written informed consent was obtained from all patents of all infants before the study.

Study group inclusion criteria were: corrected age one year, birth weight below 1500 g and gestation age shorter than 37 weeks. All consecutive children coming for the paediatric examination at the time of corrected age one year were recruited during the period of one year.

The total number of pre-term, VLBW infants born during the recruitment period was 157. The reasons for dropout from evaluation were especially child's death, serious health complications, remote residence and parental disapproval of participation in the study. Finally 102 VLBW were evaluated.

As pre-term born infants are not fully mature at the time of birth, their chronological age does not correspond to their true biological age. Hence, a comparison with full term infants can only be made if the age of pre-term infants is corrected. The corrected age was calculated by subtracting the number of weeks born before 40 weeks of gestation from the chronological age.

Control group including criteria were chronological age (a measure of an individual's age based on the calendar date on which he or she was born) one year, birth weight above 2500 g, gestation age longer, then 37 weeks and no systemic disease. These children were selected over the period of one year as they came for their first dental examination at one year of age.

Altogether 189 of one year old infants (102 VLBW and 87 NBW) were included in the study. In two cases there were twins in the study group. Information on their gestational age, mode of delivery, Apgar score (Apgar is a quick test performed on a baby at 1 and 5 min after birth. The 1-min score determines how well the baby tolerated the birthing process. The 5-min score tells the health care provider how well the baby is doing outside the mother's womb), general health status (congenital heart defects, renal and respiratory disorders, neurological disorders, vision impairments), birth weight and antibiotic use were obtained from hospital records. Feeding practice, bed-time sugar drinks and oral hygiene onset were obtained from questionnaires. All infants participated in the study were residents of communities with low natural water fluoride content (< 0.3 ppm). Children were examined clinically at the age of one year (corrected in VLBW, chronological in NBW) by two trained calibrated examiners using the dental mirror and headlight. Examiners were trained to provide treatment using the protocol approved for this study. Inter- and intra-examiner reliability was evaluated by using kappa coefficients before the start of the study. The standard methodology recommended by World Health Organization has been used (https://apps.who.int/iris/handle/10665/66783). The dental examiners were blinded to the group of infants. The number of erupted teeth (if any part of its crown had penetrated the oral mucosa), developmental defects of enamel (DDE) based on DDE criteria and hard palate deformities were evaluated [8]. The morphology assessment of the infant palate was visual using the non-standardized visual and metrical measurements, because the adequate three-dimensional shape methodology has not been developed [9].

Samples for microbial analysis.

Samples for microbial evaluation were taken from both children (91 VLBW and 86 NBW) and mothers (89 VLBW and 86 NBW). In two cases of the VLBW study group there were twins. Some samples were collected in insufficient quantities for microbial and serotype analysis, therefore there were less samples then primary included individuals. Unstimulated saliva samples from the dorsum of the tongue of children and their mothers were collected with a sterile cotton swab. Each swab was transferred into a sterile tube for the detection and identification of the main known cariogenic pathogens *Streptococcus mutans, Streptococcus sanguinis, Streptococcus salivariu and Streptococcus viridans.* Samples for the detection of cariogenic bacteria were inoculated on the selective diagnostic media Mitis Salivarius Agar (OXOID, UK) and incubated 48 h in 37 degree of Celsius at atmosphere with 10% CO_2_. The main characteristics of the cariogenic bacteria were identified (STREPTOtest 24, ERBA-LACHEMA). Serotypes of the isolated *Streptococcus mutans* were detected using the latex agglutination test Prolex Streptococcal Grouping Latex Kit (BioVendor-Laboratorní medicína a.s, Czech Republic) according the manufacturer instructions. 74 isolates from children and 90 isolates from mothers (both VLBW and control group) were used for the serotyping.

Statistical analyses.

Data were described by absolute and relative frequencies of categorical variables and median of quantitative variables. For comparison of categorical variables in groups chi-square test was used, in case of quantitative variables the Mann Whitney *U* test was adopted, kappa statistics was used for correlation. Analysis was performed in the Stat Graphics Software (Stat Point Technologies, Inc. Warrenton, Virginia, USA), MedCalc v. 9.5.2.0 (MedCalc Software, Belgium) and Microsoft Excel 2016 (Microsoft, USA). Statistical significance was set at p < 0.05 two sided.

## Results

### Anamnestic data of the newborns (study group VLBW and control group NBW)

The total number of 189 infants was included in the study, 102 VLBW and 87 NBW. VLBW newborns (the infant from the delivery up to first 28 days of life) had statistically significant shorter gestation age, 29.6 weeks compared to NBW newborns, 39.8 weeks (p < 0.0001) and significantly higher incidence of operation delivery (Caesarean section), 88.2% in comparison to control group, 11.8% (p < 0.0001). The mean birthweight of VLBW children was 1224 g and they expressed the lower value of Apgar score. The mean birthweight of NBW children was 3315 g. The differences were statistically significant (p < 0.0001).

### Anamnestic data and oral conditions of one year old children

One year old pre-term children significantly more suffered from systemic diseases compared to control group (p < 0.0001). The most frequent systemic diseases were congenital heart defects (9.8%), renal and respiratory disorders (7.9%), neurological disorders (2%) and visual impairments (1%). Systemic diseases in NBW children were diagnosed only in 3 cases (atopic eczema, congenital heart defect). Statistical significance has been found also in regular medication (p = 0.007). Vitamin D has not been considered as regular medication. Infant characteristics comparison between VLBW and NBW group is presented in Table 1.

**Table 1:** Infant characteristics comparison between VLBW and NBW group

		VLBW		NBW		p value*
Number		102		87		
Gestational age [weeks median, (IQR)]		29.6	(27.3–31.7)	39.8	(38.4–40.1)	**p < 0.001**
Birth weight [grams median, (IQR)]		1224	(860–1410)	3315	(3120–3700)	**p < 0.001**
Apgar score [number median, (IQR)]	At 1 min	7	(6–8)	10	(9–10)	**p < 0.001**
	At 5 min	8	(7–9)	10	(10–10)	**p < 0.001**
	At 10 min	8,5	(8–9)	10	(10–10)	**p < 0.001**
Mode of delivery [N, (%)]	Vaginal	12	(11.8)	71	(81.6)	**p < 0.001**
	Caesarean section	90	(88.2)	16	(18.4)	**p < 0.001**
Antibiotic treatment [N, (%)]	AMPI	69	(67.6)	0	(0.0)	**p < 0.001**
	AMPI + surfactant	59	(57.8)	0	(0.0)	**p < 0.001**
	GENTA	66	(64.7)	0	(0.0)	**p < 0.001**
	Other	48	(47.1)	0	(0.0)	**p < 0.001**
Infection [N, (%)]	Early infection	50	(49.0)	0	(0.0)	**p < 0.001**
	Late infection	38	(37.3)	0	(0.0)	**p < 0.001**
General diseases [N, (%)]	Present overall	24	(23.5)	3	(3.4)	**p < 0.001**
	Bronchial asthma	4	(3.9)	0	(0.0)	p = 0.06
	Atopic eczema	0	(0.0)	2	(2.3)	p = 0.121
	Respiratory disorders	7	(6.9)	0	(0.0)	**p = 0.011**
	Heart defects	10	(9.8)	1	(1.1)	**p = 0.009**
	Renal disorders	1	(1.0)	0	(0.0)	p = 0.353
	Neurological disorders	2	(2.0)	0	(0.0)	p = 0.187
	Visual impairment	1	(1.0)	0	(0.0)	p = 0.353
Medication [N, (%)]	Regular use	8	(7.8)	0	(0.0)	**p = 0.007**

IQR interquartile range, N number, VLBW very low birth weight, NBW normal birth weight, AMPI ampicillin, GENTA gentamicin

*Statistical significance determined using ML chi-square test in case of categorical variables and Mann–Whitney *U* test in case of continuous variables, significant values marked in bold

VLBW children got more frequently sweetened drinks during the day and night comparing to control NBW children. The difference was statistically significant (p = 0.007). Regular oral hygiene practice was more frequent in full term control group (p = 0.002). Significantly more NBW full term children were breast fed frequently at night when compared to VLBW pre-term children (p < 0.001). This can be explained by the fact that VLBW children were hospitalized for several months at the intensive care units, had less frequent contact with mother, antibiotic therapy and parenteral nourishment.

Significant difference has been found between VLBW and NBW children also in number of erupted teeth, when VLBW children had less erupted teeth compared to control group of children (p = 0.036). Developmental defects of enamel were recorded in both groups, but more frequently in premature children In case of enamel hypoplasia (20.6%) the difference was statistically significant (p = 0.033), no significance (p = 0.0736) was found in case of hypomineralization (10.8%). Both hypoplasia and hypomineralization had association with prematurity and children with such defects had significantly lower birth weight when compared to children without developmental defects. Oral characteristics and comparison between VLBW and NBW group is summarised in Table 2.

**Table 2:** Oral characteristics and comparison between VLBW and NBW group

	VLBW		NBW		p value*
Number	102		87		
Oral hygiene [N, (%)]	76	(74.5)	72	(82.8)	**p = 0.002**
Sweetened drinks [N, (%)]	39	(38.2)	20	(23.0)	**p = 0.007**
Night breast-feeding [N, (%)]	6	(5.9)	31	(35.6)	**p < 0.001**
Erupted teeth [number median, (IQR)]	6	(4–8)	7	(5–8)	**p = 0.036**
Hypoplasia [N, (%)]	21	(20.6)	2	(2.3)	**p = 0.033**
Hypomineralization [N, (%)]	11	(10.8)	3	(3.4)	p = 0.0736
Hard palate deformities [N, (%)]	12	(11.8)	0	(0.0)	**p < 0.001**

IQR interquartile range, N number, VLBW very low birth weight, NBW normal birth weight

*Statistical significance determined using ML chi-square test in case of categorical variables and Mann–Whitney *U* test in case of continuous variables, significant values marked in bold

### Microbial findings

Representation of streptococcal species among infants and mothers are presented in Table 3. Proportional representation of *S. mutans* after the cultivation (MSA + bacitracine) did not reveal statistical difference in both groups of children (p = 0.484). No statistical difference has been found in proportional representation of *S. mutans* and other streptococci between mothers of full-term infants and mothers of pre-term infants (p = 0.385). Lower proportion of *S. mutans* was compensated by the higher proportion of *S. sanguis* and *S. viridans*.

**Table 3:** Representation of streptococcal species among infants and mothers

	Infants		p value*	Mothers		p value*
	VLBW	NBW		VLBW	NBW	
*Streptococus mutans* [% median, (IQR)]	0.0 (0.0–3.5)	0.7 (0.0–5.0)	0.484	15.7 (8.8–22.0)	15.0 (9.0–19.0)	0.385
*Streptococus sanguis* [% median, (IQR)]	32.0 (27.0–38.6)	35.4 (30.9–41.0)	**0.047**	29.7 (21.5–35.5)	30.1 (25.3–35.3)	0.535
*Streptococus viridans* [% median, (IQR)]	38.8 (31.7–48.7)	39.1 (28.8–46.6)	0.601	33.0 (25.4–42.1)	34.7 (27.4–44.0)	0.439
*Streptococus salivarius* [% median, (IQR)]	19.7 (12.0–25.0)	16.6 (11.9–20.6)	0.130	15.0 (10.0–20.0)	15.7 (11.5–20.5)	0.870
Other *streptococci* [% median, (IQR)]	2.0 (0.0–5.8)	2.5 (0.0–5.8)	0.819	1.3 (0.0–5.2)	1.6 (0.0–6.7)	0.995

IQR interquartile range, N number, VLBW very low birth weight, NBW normal birth weight

*Statistical significance determined using Mann–Whitney *U* test, significant values marked in bold

Pre-term children had significantly lower prevalence of *S. mutans* (p = 0.0002) and marginally significant low grow density (p = 0.0512), Table 4.

**Table 4:** Prevalence and density of S.mutans in children with VLBW and LBW

	N	Prevalence		Density
		N pos	%	%
VLBW	91	12	13.19	2.2
NBW	86	42	48.84	8.1
		p = 0.0002*	p = 0.0512*	

VLBW very low birth weight, NBW normal birth weight

*Statistical significance determined using ML chi-square test, significant values marked in bold

Almost 40% of isolates *S. mutans* have shown one from the group anti-gens c, e, f or k. 60% of cases were undetectable. The most frequently detected was serotype c (68.89% in mothers and 67.57% in children), Table 5. The correlation of serotypes of *S. mutans* isolates has been evaluated and was found 92.52% in serotype c, 87.56% (e), 66.67% (f) and 83.33% (k), Table 6. The correlation of serotypes of *S. mutans* strains isolated from mothers and their children was κ = 0.832).

**Table 5:** Serotypes of S.mutans in children and their mothers

S. mutans serotypes	Mothers		Children	
Frequency	N	%	N	%
c	62	68.89	50	67.57
e	16	17.77	8	10.81
f	6	6.67	10	13.51
k	6	6.67	6	8.11
	p = 0.3308*			

VLBW very low birth weight, NBW normal birth weight

*Statistical significance determined using ML chi-square test, significant values marked in bold

**Table 6:** The correlation of S. mutans serotypes in children and mothers

	Serotype	S. mutans isolates from mothers							
		c		e		f		k	
correlation		N	%	N	%	N	%	N	%
S. mutans isolates from children	c	50	92.59	0		2		1	
	e	0		14	87.50	0		0	
	f	0		2		4	66.67	0	
	k	2		0		0		5	83.33

## Discussion

One year old pre-term children in this study suffered significantly more from systemic diseases compared to control group, what may effect orofacial development and oral health. Burt and Pai have reported that pre-term birth and low birth weight are closely related to increased risk of early and late infant morbidity including dental caries in early childhood [10]. Frequent consumption of sweetened drinks, night breast feeding on demand and late onset of oral hygiene practice are considered as other important caries risk factors. VLBW children in our study got significantly more frequently sweetened drinks during the day and night comparing to control NBW children. Davenport et al. have reported a greater sugar intake, and consequently greater number of dental caries in pre-term, VLBW children comparing to full term children [5]. Significantly more NBW full term children in this study were breast fed frequently at night when compared to VLBW pre-term children. This can be explained by the fact that VLBW children were hospitalized for several months at the intensive care units, had less frequent contact with mother, antibiotic therapy and parenteral nourishment. Regular oral hygiene practice was more frequent in full term control group, but with no statistical significance. However predisposition to dental caries in premature children is somewhat controversial, because of the scarcity of studies and necessity to evaluate other primary and secondary risk factors as well as socio-economic consequences [10, 11]. One study by Schüler et al. found an association of dental caries with low socio-economic status [12].

VLBW is closely associated with delayed tooth eruption [13]. Viscardi et al. [14] have found that primary teeth eruption occurred significantly later in infants with birth weight < 1000 g and gestation age ≤ 30 weeks. Seow et al. [15] have reported that VLBW infants have less erupted teeth compared to NBW peers at their chronological age 6–17 month. The results of recent study also confirmed the significant lower number of erupted primary incisors in VLBW children compared to NBW children.

DDE are well studied complications of prematurity and may be present as enamel hypoplasia or enamel hypomineralization [16]. The possible pathogenesis of DDE in pre-term children may be explained by the presence of many systemic disturbances in prenatal and perinatal period (metabolic disorders, respiratory infections, nutritional disorders as vitamin D and calcium deficiency, birth asphyxia and respiratory distress [3, 17]. In our study, enamel hypoplasia has been found in 20.6% and hypomineralization in 10.8% of VLBW children. Enamel hypoplasia with porous and rough surface, combined with feeding problems, frequent consumption of sugar containing diet, behavioural disorders and other risk factors, predispose the pre-term, VLBW children to dental caries in early age [18]. An association of LBW and enamel defects was described by Cortines et al. In 2019 [19].

The composition of oral bacteria population in healthy full-term children and their mothers were published. The authors evaluate the microbial colonisation of oral cavity in children, transmission of cariogenic bacteria and the role of *S. mutans* in aetiology of early childhood caries [20, 21]. These factors are insufficiently concerned in pre-term, VLBW children. Makhoul et al. [22] investigated 65 pre-term children and found significant rise in bacterial colonization from the first up to 10th day of life regardless of gestation age and antibiotic therapy. The spectrum of microbial flora is chancing from the 10th day due to antibiotic therapy with the dominance of coagulase negative staphylococci. The intensity of microbial harbouring of oral cavity in newborns is affected by mucosal immunity system, gestational age, mode of delivery, intensive care unit hospitalization, feeding practice and antibiotic therapy in neonatal period. Based on our findings, VLBW children had significantly more antibiotic medication comparing to control group. Microbial acquisition of oral cavity might be delayed due to surgical mode of delivery and parenteral nourishment [23, 24]. It is in concordance with our findings. NBW children from the control group section delivery had significantly lower prevalence of *S. mutans* when compared to vaginal delivery.

The early infection of mutans streptococci is associated with caries risk in young children. It was assumed that infants acquire mutans streptococci from their mothers after the eruption of primary teeth. More recent studies have reported that these bacteria can colonize the mouth of pre-dentate infants. The Swedish study by Nelun Barfod [25] has revealed the 60% detection rate of *S. mutans* in children aged from 6 to 10 months. Our findings closely correlate with Australian study using the quantitative RT PCR technique [26]. The differences in detection of oral streptococci may be explained by different laboratory methods used in individual studies.

The studies [26, 27] evaluating the oral bacterial colonization in VLBW and NBW children have focused mainly on *S. mutans*. Wan et al. [6] detected S. mutans in 60% of 6 month old full-term infants and in 50% of the same aged pre-term infants. The same author has found the increasing presence *S. mutans* with age. *S. mutans* was detected in 37% of one year old children with higher prevalence in full-term children compared to pre-term children. In this study, *S. mutans* was detected in 13.19% of VLBW children and in 48.84% of NBW children. Pre-term children had significantly lower prevalence of *S. mutans* and marginally significant low grow density. Considering the age of examined children (one year), our detection rate is higher compared to Plonka et al. [28] and lower compared to Merglova et al. [29]. She found the presence of *S. mutans* in 4.2% of VLBW children and in 100% of NBW children. These observations, coupled with the finding of Fujiwara et al. [30] and Roeters et al. [31] clearly illustrate that the presence of *S. mutans* at one year of age is the effective caries predictor in early childhood. Our study did not reveal differences in *S. mutans* in VLBW and NBW infants therefore it cannot be concluded that possible higher risk of caries later is attributed to *S. mutans*. Similarly, Japanese study by Tanaka [32] did not find any difference in caries prevalence in LBW children. The frequent consumption of sweetened drinks during the day and mainly at night and high salivary levels of cariogenic bacteria in mothers significantly contribute to *S. mutans* colonization of their children [33]. Mothers of both groups of children in our study did not significantly differ neither in the prevalence nor in the grow density of *S. mutans*. The low prevalence and late *S. mutans* colonization in pre-term, VLBW children can be explained by less frequent contact between mother and infant due to long term hospitalization of these children at intensive care unit, antibiotic therapy, parenteral nourishment and endo-tracheal intubation.

There are some limitations of the study. One of the possible limitations of the study is the fact that the dropout of VLBW children reached 35% from the intended population which is the fact that may produce a bias in the result interpretation. Aetiology of dental caries is multifactorial and not all risk factors have been evaluated in this study. Systemic diseases in early childhood have not the same impact on caries risk, although the systemic disease is generally considered as the risk factor. The most important is the subsequent parental attitude to oral health and dental care. The further long term observation of children with VLBW and prematurity is necessary for better identification the caries risk factors.

## Conclusion

The study confirmed anamnestic and medical differences between one year old pre-term, VLBW infants and full-term NBW infants. The proportional representation and count of *S. mutans* did not reveal statistical difference neither in VLBW and NBW children, nor in their mothers. Dental health instructions, setting of appropriate dietary and oral hygiene habits, as well as the adoption of efficient preventive measures are of paramount importance in VLBW babies to prevent dental problem in childhood.

## Data Availability

The datasets used and/or analysed during the current study available from the corresponding author on reasonable request.

## Abbreviations

AMPI: Ampicillin
DDE: Developmental defects of enamel
GENTA: Gentamicin
IQR: Interquartile range
LBW: Very low birth weight
N: Number
NBW: Normal birth weight
VLBW: Very low birth weight
